# An Entropy-Based Anti-Noise Method for Reducing Ranging Error in Photon Counting Lidar

**DOI:** 10.3390/e23111499

**Published:** 2021-11-12

**Authors:** Mingwei Huang, Zijing Zhang, Jiaheng Xie, Jiahuan Li, Yuan Zhao

**Affiliations:** School of Physics, Harbin Institute of Technology, Harbin 150001, China; 18B911040@stu.hit.edu.cn (M.H.); xiejiaheng@hit.edu.cn (J.X.); lijiahuan@hit.edu.cn (J.L.)

**Keywords:** entropy, photon counting lidar, noise mitigation, ranging error

## Abstract

Photon counting lidar for long-range detection faces the problem of declining ranging performance caused by background noise. Current anti-noise methods are not robust enough in the case of weak signal and strong background noise, resulting in poor ranging error. In this work, based on the characteristics of the uncertainty of echo signal and noise in photon counting lidar, an entropy-based anti-noise method is proposed to reduce the ranging error under high background noise. Firstly, the photon counting entropy, which is considered as the feature to distinguish signal from noise, is defined to quantify the uncertainty of fluctuation among photon events responding to the Geiger mode avalanche photodiode. Then, the photon counting entropy is combined with a windowing operation to enhance the difference between signal and noise, so as to mitigate the effect of background noise and estimate the time of flight of the laser pulses. Simulation and experimental analysis show that the proposed method improves the anti-noise performance well, and experimental results demonstrate that the proposed method effectively mitigates the effect of background noise to reduce ranging error despite high background noise.

## 1. Introduction

Benefiting from the excellent photon-efficiency sensitivity and picosecond-level temporal resolution, photon counting lidar equipped with Geiger mode avalanche photodiode (Gm-APD) has been widely used in long-range high-resolution ranging and 3D imaging [1,2,3,4,5,6,7]. Ranging error is the most important index to evaluate the ranging performance of photon counting lidar. One of the main factors that introduce ranging error is inevitable noise such as solar light during detection. Theoretical analysis has predicted that the ranging error increases with the increase of noise [8]. Therefore, it is essential to deal with noise before extracting the ranging signal.

Several anti-noise methods have been proposed for photon counting lidar. In ranging applications, a routinely used method is the iterative sigma multiplier filter. It cuts off the photon counting histogram within a region near the signal peak with the width of a multiplier of the emitted pulse’s standard deviation [9,10]. Its reliability is very dependent on the accuracy of the peak position of the echo signal. Another widely used method is to set a threshold to filter noise. A coincidence processing of multiple pulses with the aid of a threshold algorithm has been proposed to discriminate noise to improve the detection probability of the Gm-APD detector [11]. Zhang et al. proposed an adjacent threshold method to filter noise, and obtained the depth image of target in noisy condition [12]. The direct thresholding method is convenient and can achieve good performance when the signal-to-noise ratio (SNR) is relatively high, but when SNR decreases, it is likely to misjudge noise counts into signal counts. In 3D imaging applications, some anti-noise methods are also proposed before signal extraction. Based on the hypothesis that a cluster in the photon counting histogram is due to signal, Joshua Rapp et al. proposed a noise censoring method [13], and Hua et al. proposed a first signal photon unit method [14] to discriminate signal counts to obtain clearer photon counting data. Such methods are applicable in low flux scenes, but the hypothesis is not satisfied in high background scenes. The mentioned anti-noise methods can be classified as histogram-based methods, but they are affected strongly by background noise.

The more effective method to deal with noise is a correlation-based method that is called matched filter, which performs a cross-correlation between the system response with the photon counting histogram to extract the signal. Aurora et al. used a matched filtering method to suppress the background noise in a highly scattering environment, and estimate the depth image of the underwater target [15]. Markus et al. performed matched filtering to the photon counting histograms to improve the characteristics of the signal for determining the positions and amplitudes of the echo peaks [16]. The matched filter works at a lower SNR than the histogram-based methods, however, under weak signal and strong noise condition, it gives several extrema and the location of the signal peaks is hard to determine [17]. Besides, when the echo waveform is affected by the detection scene, it is necessary to measure the impulse response of the real-time scene in advance, but it is difficult to achieve under a strong background.

In summary, both the histogram-based method and correlation-based method lack enough robustness under a weak signal but strong background noise cases, such as the scenes of long-range detection in daytime, resulting in poor ranging error. Therefore, a more robust anti-noise technology needs to be established. Entropy is an important tool to quantitatively evaluate the uncertainty of random events, and recently many technologies using entropy to improve detection performance have been developed in radar, ultra-wide bandwidth ranging and location, fuzzy clustering, and cypher system [18,19,20,21]. For the range measurement process of photon counting lidar, noise is a stationary random process and signal is a nonstationary random process, which means that the fluctuation of noise photon events (PEs) is completely random and that of signal PEs is related to laser pulse, i.e., the uncertainty of the fluctuation among noise events is much higher.

In this paper, based on the mentioned characteristic, an entropy-based method to mitigate the effect of background noise is proposed to reduce the ranging error in photon counting lidar. Firstly, the photon counting entropy is defined as a statistical test to quantify the uncertainty of fluctuation among the PEs responded by Gm-APD. Then, a window is used to divide the sampling period into multiple subintervals, and the photon counting entropy is calculated in each subinterval as the feature that differs signal and noise. Thirdly the subinterval with the minimum photon counting entropy is searched, whose time delay is the estimation of the time of flight. The simulation and experiment show that the proposed method effectively deals with noise to reduce ranging error despite high background noise. Under a background noise rate of 9 MHz, for example, range accuracy and range precision of the proposed method are both about five times better than those of the matched filter method.

## 2. Anti-Noise Method Based on Photon Counting Entropy

### 2.1. The Photon Counting Entropy

**Figure 1:**
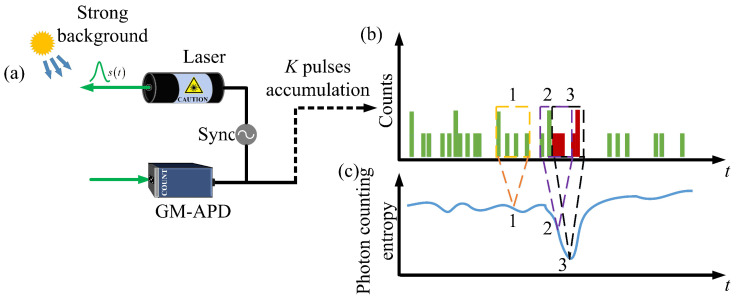
Signal characteristic analysis in photon counting lidar. (**a**) The typical model of a photon counting ranging system, which is mainly composed of the pulsed laser, the Gm-APD and the time correlator; (**b**) The schematic of photon counting histogram with *K* pulses accumulation, in which the green columns represent the noise and the red columns represent the signal; (**c**) The schematic of qualitatively depicting photon counting entropy among detected PEs, the dashed box with numbers ‘1’, ‘2’, and ‘3’ represent the quantified uncertainty among noise PEs, some signal PEs mixing with some noise PEs, and signal PEs, respectively.

The characteristic of echo signal in photon counting lidar is firstly analyzed. As shown in Figure 1a, a typical photon counting ranging system uses a pulsed laser to periodically illuminate the target, and uses a Gm-APD to respond the individual PEs that are caused by the backscattered signal from the target plus the background noise and dark noise. A time correlator generates time stamps of the photon avalanche events and builds a photon arrival time histogram. With multiple pulses accumulation, the photon counting histogram shown in Figure 1b is obtained, in which the red and green columns represent signal PEs and noise PEs, respectively. For a system with typical parameters at 532 nm, with a 1 nm narrowband optical filter, a 10 cm receiver aperture and a 100 μrad receive field of view, the solar background noise rate is estimated to be about 10 MHz in daytime [22]. The system is affected strongly by the solar background, especially for long dead time detectors, as at most one avalanche event can occur per emitted laser pulse. In this paper, the photon counting entropy is proposed to exploit the difference in uncertainty between signal and noise for mitigating the noise effect. Due to the stationary characteristic of the noise and the nonstationary characteristic of the signal, the uncertainty of the fluctuation among noise PEs is much higher than that of the signal PEs. Figure 1c qualitatively depicts the photon counting entropy among detected PEs, where the dashed box with numbers ‘1’, ‘2’, and ‘3’ represent the quantified uncertainty among noise PEs, some signal PEs mixing with some noise PEs, and signal PEs, respectively.

According to Poisson statistic [23], for long dead time case, the mean PEs’ counts in the *i*-th time bin is derived after *K* pulses accumulation as

(1) $$\bar{y}\left(i\right)=K\exp\left[-\int_{0}^{(i-1)\Delta t}\lambda_{\mathrm{sn}}\left(t\right)dt\right]\left\{1-\exp\left[-\int_{\left(i-1\right)\Delta t}^{i\Delta t}\lambda_{\mathrm{sn}}\left(t\right)dt\right]\right\},\;\;i=1\cdots N,$$

where $\lambda_{\mathrm{sn}}\left(t\right)=\lambda_{s}\left(t\right)+\lambda_{n}\left(t\right)$ is the rate function of total echo photoelectrons at time *t*, in which $\lambda_{s}\left(t\right)$ is the signal rate, and $\lambda_{n}\left(t\right)$ is the total noise rate of the summation of background noise and dark counts, Δt is the time resolution of the time correlator, and *N* is the number of time bins. The detected PEs’ counts $y\left(i\right)$ in the *i*-th bin fluctuates around Equation (1). The proposed photon counting entropy exploits the stationary characteristic of fluctuation among neighbor time bins to evaluate the difference in signal and noise. Assuming that the noise rate is estimated as ${\hat{\lambda }}_{n}\left(t\right)$, the random fluctuation of the detected counts $y\left(i\right)$ in the *i*-th bin is estimated by

(2) $$\hat{y}\left(i\right)=y\left(i\right)-K\exp\left[-\int_{0}^{\left(i-1\right)\Delta t}{\hat{\lambda }}_{n}\left(t\right)dt\right]\left\{1-\exp\left[-\int_{\left(i-1\right)\Delta t}^{i\Delta t}{\hat{\lambda }}_{n}\left(t\right)dt\right]\right\}.$$

The fluctuation of noise PEs’ counts is mainly determined by the probabilistic response of the background noise. The solar background is a thermal noise source, and the fluctuation of noise PEs’ counts at each time bin is statistically independent. Thus, the distribution of noise fluctuations has the characteristics of white noise approximately, and its power spectrum is uniformly distributed. The distribution of signal fluctuation is more affected by the fluctuation in number of signal photoelectrons, and it is related to the laser pulse. Therefore, they are distinguished in the spectral domain. The discrete Fourier transform is performed to Equation (2)

(3) $$\hat{Y}\left(k\right)=\sum_{i=1}^{N}\hat{y}\left(i\right)\exp\left(-j2\pi ki/N_{k}\right),$$

where $\hat{Y}\left(k\right),\;k=1,2,\cdots ,N_{k}$ is the *k*-th sampling point in Fourier domain of $\left\{\hat{y}\left(i\right)\right\},\;i=1,2,\cdots ,N$, and $N_{k}$ is the number of discrete frequency points. Since the entropy of uniform distribution maximizes and that of other distribution reduces, the proposed photon counting entropy is defined in the spectral domain to evaluate the uncertainty of the fluctuation among PEs. Using Equation (3), the photon counting entropy is defined as

(4) $$H_{c}=-\sum_{k=1}^{N_{k}}\frac{{\left|\hat{Y}\left(k\right)\right|}^{2}}{\sum_{k=1}^{N_{k}}{\left|\hat{Y}\left(k\right)\right|}^{2}}\ln\frac{{\left|\hat{Y}\left(k\right)\right|}^{2}}{\sum_{k=1}^{N_{k}}{\left|\hat{Y}\left(k\right)\right|}^{2}},$$

where ${\left|Y\left(k\right)\right|}^{2}$ denotes the power of *k*-th discrete frequency point. If only noise exists during detection, according to the above analysis, the fluctuation among PEs’ counts is approximately white noise with uniform power spectrum, and the ideal photon counting entropy of noise counts is then

(5) $${H_{c}}_{,\;\mathrm{noise}}\approx \ln N_{k}.$$

if only the signal exists during detection, the fluctuation among PEs’ counts is approximate to a Gaussian function with multiple pulses accumulation [24]. Here a Gaussian function is used to approximate the fluctuation among PEs’ counts, and according to Equations (3) and (4), the photon counting entropy of signal counts is

(6) $${H_{c}}_{,\;\mathrm{signal}}\approx \frac{1}{2}\ln\frac{\sqrt{2}eN_{k}}{\Delta \sigma },$$

where Δσ=σ/Δt denotes the discrete standard deviation of laser pulse. For the detection process, it often satisfies Δσ≫1 and $N_{k}\gg \sqrt{\sqrt{2}eN_{k}/\Delta \sigma }$, and thus the magnitude of ${H_{c}}_{,\;\mathrm{signal}}$ is significantly less than that of ${H_{c}}_{,\;\mathrm{noise}}$. Actually, signal and noise both exist during detection. With the number of signal photoelectrons increasing, the influence of noise on the fluctuation of detected counts reduces, and photon counting entropy reduces.

### 2.2. The Proposed Anti-Noise Method

**Figure 2:**
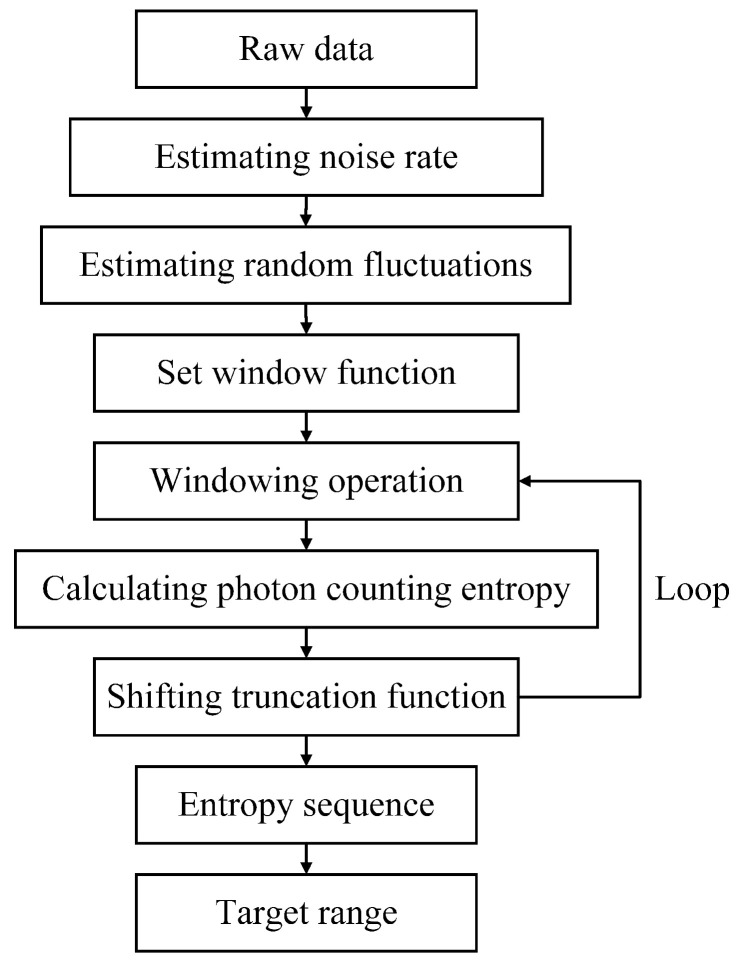
The flow chart of the anti-noise method based on photon counting entropy.

Based on the defined photon counting entropy and its difference between noise and signal, a method combining photon counting entropy and windowing operation is proposed to mitigate the effect of background noise and reduce ranging error. The flow chart of the proposed method is illustrated in Figure 2. Firstly, the noise rate is estimated using the raw photon counting data, and the processing described by Equation (2) is performed to estimate the fluctuation in each time bin. Then, a window function is used to divide the sampling period into multiple subintervals, and the photon counting entropy is calculated as the feature that evaluates the uncertainty of fluctuation among photon counts in each subinterval. With the window function shifting, a sequence of photon counting entropy is obtained to replace the raw photon counting data to describe the echo characteristics. Finally, the subinterval with the minimum photon counting entropy is searched, and the time delay of the searched subinterval is regarded as the estimation of the time of flight.

#### 2.2.1. Estimating Noise Rate

The noise rate is firstly estimated using the raw photon counting data. As noise rate is uniform in the sampling period, and the target is not usually located at the front of the sampling period, it is reasonable to assume that only noise PEs’ counts are included in the first *x* time bins. Thus, the noise rate during detection is expressed as, according to Poisson statistics [23],

(7) $${\hat{\lambda }}_{n}\left(t\right)=-\frac{1}{x}\log\left(1-\frac{1}{K}\sum_{i=1}^{x}\bar{y}\left(i\right)\right),$$

where *K* is the number of the accumulated laser pulses. Equation (7) is true only if *K* is large enough, and it often satisfies in weak signal but strong noise condition, as several thousands of pulses are necessary in this case. In order to ensure that the noise rate is correctly estimated and no signal is included in the first *x* time bins as much as possible, x=50 is chosen in this paper.

#### 2.2.2. Window Function

The sampling period is divided into multiple subintervals by a window function. Since photon counting entropy is defined in spectral domain, the window has an effect on the spectrum of the preprocessed data. In order to avoid this, the hamming window that has the lowest sidelobe level is chosen to divide the sampling period. The hamming window refers to the window that satisfies the following function

(8) $$w\left(m\right)=\left\{\begin{array}{cc} 0.54-0.46\cos\frac{2\pi m}{M-1} & ,0\leq m\leq M-1\\ 0 & else \end{array}\right.,$$

where *M* is the window width. The window width is an important parameter that affects anti-noise performance. If *M* is too small, the stationarity of the noise is not satisfied as it must be observed over a relatively long time. If *M* is too large, the nonstationarity of the signal may not be distinguishable to two adjacent ranges. After a lot of trial and error, the proper width is set as M=6.5σ to obtain the best performance, where σ is the standard deviation of laser pulse. A simulation is detailed in the Appendix A to demonstrate that M=6.5σ is a proper parameter.

#### 2.2.3. Noise Mitigation

**Figure 3:**
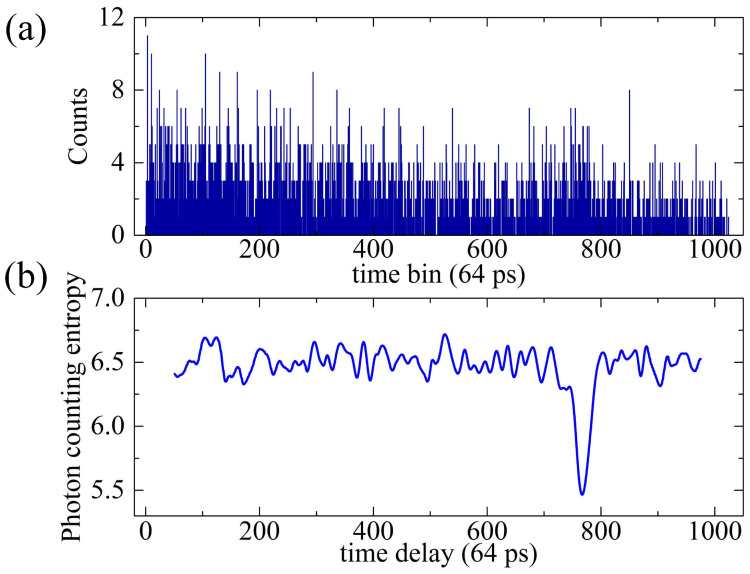
Simulation of mitigating the effect of background noise. (**a**) Raw photon counting histogram under weak signal but strong background noise; (**b**) The noise mitigation result after replacing the raw data with the sequence of photon counting entropy.

The photon counts of the windowed *q*-th subinterval is expressed as

(9) $${\hat{y}}_{q}\left(m\right)=w\left(m\right)\hat{y}\left(m+qs\right),\;q=0,1,\cdots ,\;Q-1,$$

where *s* denotes the shifting step between two adjacent window operations, and *Q* is the number of truncated subintervals and Q=(N−M−s)/s. In order to take full advantage of the time resolution of time correlator, s=1 is set. Substituting Equation (9) into Equations (3) and (4), the photon counting entropy $H_{c,q}$ of *q*-th subinterval is obtained. With the window function shifting, the sequence of photon counting entropy is

(10) $$H_{c}=\left[{H_{c,\;}}_{1},\;\;{H_{c,\;}}_{2},\;\;\;\cdots ,\;\;\;\;{H_{c,\;}}_{Q}\;\right],$$

which indicates the trend of how the uncertainty of fluctuation among PEs’ counts varies with the time axis. The sequence of photon counting entropy replaces the raw data to describe the echo characteristics and mitigate the effect of noise. To verify the feasibility of the proposed method, the simulation shown in Figure 3 is taken. Under weak signal and strong background noise condition ($N_{s}=0.05,N_{n}=1$ in the sampling period and K=4000, the signal to noise ratio [15] $\mathrm{SNR}=\sqrt{K}N_{s}/\sqrt{N_{s}+N_{n}}$ is 7.7, the photon counting histogram is simulated as shown in Figure 3a, where the signal is not distinguishable, and the corresponding sequence of photon counting entropy is shown in Figure 3b, where the difference between signal and noise is enhanced significantly. Therefore, the effect of noise is effectively mitigated by replacing the raw photon counting data with the sequence of photon counting entropy.

#### 2.2.4. Range Estimation

The time of flight is estimated as the time delay of the subinterval with minimum photon counting entropy. It satisfies the condition of

(11) $$q_{0}=\arg\;\;\;\min\left[{H_{c,\;}}_{1},\;\;{H_{c,\;}}_{2},\;\;\;\cdots ,\;\;\;\;{H_{c,\;}}_{Q}\;\right],\;\;\;\;s.t.\;\;\;\;{\left.\frac{\partial H_{c}}{\partial q}\right|}_{q=q_{0}}=0,$$

with which the local minima is searched by peak searching to the sequence of negative photon counting entropy $-H_{c}$. Then, the time delay of the $q_{0}$-th subinterval is expressed as

(12) $$t_{q_{0}}=\frac{M}{2}\Delta t\;+\left(q_{0}-1\right)s\Delta t\;,$$

where Δt is the time resolution of the time correlator. The range of the target is thus

(13) $$R=\frac{1}{2}ct_{q_{0}},$$

in which *c* is the speed of light.

### 2.3. Ranging Performance Evaluation

Range accuracy and range precision are two indicators often used to quantitatively evaluate ranging error. Range accuracy represents the systematic errors [25]. It is defined as

(14) $$\sigma_{\mathrm{accuracy}}=\frac{1}{L}\sum_{l=1}^{L}\left|R_{l}-R_{\mathrm{real}}\right|,$$

where $R_{\mathrm{real}}$ is the true value of the target range, $R_{l}$ is the measured range of *l*-th measurement, and *L* is the number of measurements. Range precision represents the random errors of system [25]. It is defined as the statistical variance of the distribution of measured ranges, i.e.,

(15) $$\sigma_{\mathrm{precision}}=\sqrt{\frac{1}{L}\sum_{l=1}^{L}{\left(R_{l}-\bar{R}\right)}^{2}},$$

where $\bar{R}$ is the mean value of multiple ranging measurements.

Finally, considering that incorrect range estimations may exist because of high background noise, another quantitative indicator named the correct ranging rate is defined to evaluate the robustness of range estimation. Those range estimations distributed in the region $\left[R_{\mathrm{real}}-3\sigma ,R_{\mathrm{real}}+3\sigma \right]$ are defined as correct estimations, where σ is the standard deviation of laser pulse, and the correct ranging rate is

(16) $$r_{c}=\frac{L_{c}}{L}$$

where $L_{c}$ is the number of the correct range estimations.

## 3. Results and Discussion

### 3.1. Simulation Analysis

In this section, combined with Equations (1) and (10)–(15), the ranging error is discussed to verify the anti-noise performance of the proposed method by simulations. The response characteristics of Gm-APD is simulated with a Monte-Carlo simulation. The parameters in the simulation are listed in Table 1. According to the radar equation [26], the signal photoelectrons $N_{s}\approx 0.05$. 2000 pulses are accumulated to obtain a photon counting histogram, and 1000 measurements are repeated in each simulation to estimate the range accuracy and range precision. The simulation performance of the proposed method is compared with the direct thresholding method and the matched filter method. For direct thresholding method, the threshold is set as a half of the maximal counts in photon counting histogram, and those counts higher than the threshold are considered as signal, and vice versa are noise. The range of target is calculated by the center of mass algorithm [26]. For the matched filter method, the Gaussian function is used as the normalized system response, and its standard deviation is set according to the pulse width. A cross-correlation is performed between the system response with the photon counting histogram to filter noise. The range of target is estimated using the location of the correlation peak.

**Figure 4:**
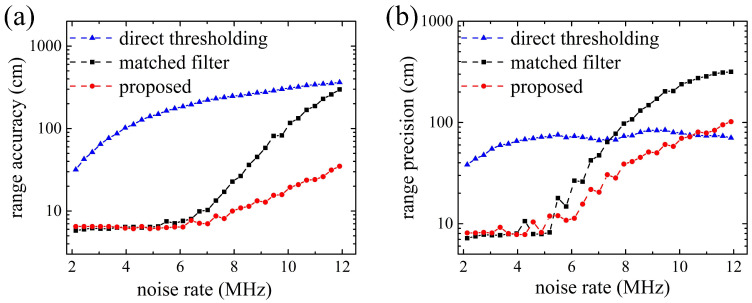
Ranging error versus background noise rate. (**a**) Range accuracy; (**b**) Range precision.

Figure 4 shows the influence of the noise level on the range accuracy and range precision of the three methods. The noise rate in the simulation ranges from 2 MHz to 12 MHz, and the corresponding SNR decreases from 8.7 to 5.9. As shown in Figure 4, the range accuracy and range precision obtained by direct thresholding filtering and center of mass algorithm estimation are always poor. The reason is that, under the simulation condition, signal and noise are hard to be distinguished by the magnitude of their counts, and in many cases some noise counts are higher than signal counts. For the other two methods, when the noise rate is low, range accuracy and precision of the two methods are both in a low level, which indicates that the proposed method filters noise as effectively as the matched filter method. However, when the noise rate is larger than 7 MHz, range accuracy and range precision of matched filter method increase rapidly from 9.6 cm and 40.5 cm to 258.2 cm and 311.1 cm, while those of the proposed method vary gently from 8.2 cm and 30.9 cm to 32.8 cm and 97.8 cm. Range accuracy and range precision of the two methods are both not convergent with increasing noise, but the proposed method maintains a ranging error better than matched filter method under the high background noise level.

**Figure 5:**
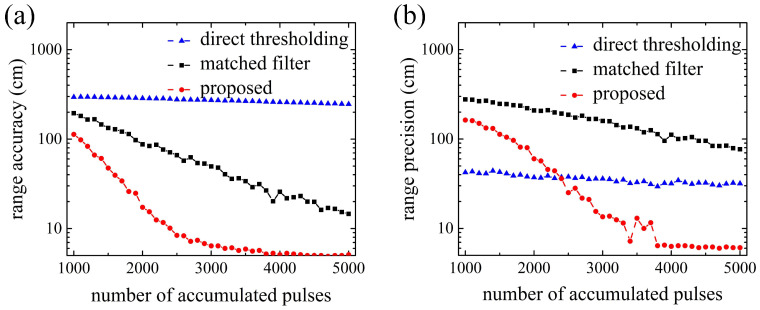
Ranging error versus the number of accumulated pulses. (**a**) Range accuracy; (**b**) Range precision.

To further reduce the ranging error under a high noise level, an effective method is to increase the number of accumulated laser pluses. Figure 5 shows the results of range accuracy and range precision varying with the number of accumulated pulses. The noise rate in simulation is 10 MHz, which is approximately the background noise level on a sunny day. As is shown in Figure 5, for the direct thresholding method, range accuracy has a slight trend to decrease with the accumulated pulses increasing, and range precision stays relatively stable, which may be because the responses of Gm-APD uniformly and randomly distribute over the time axis. A large number of the accumulated laser pulses is necessary to improve the range accuracy and range precision to the satisfied level. For the remaining two methods, range accuracy and range precision of the proposed method converge rapidly to a stable value, but those of the matched filter method do so more slowly. When accumulating 3000 pulses, SNR = 7.7, range accuracy of the proposed method stabilizes around 5.5 cm, and range precision stabilizes around 6 cm. However, the same level of range accuracy and range precision of matched filter method is not achieved until accumulating over 5000 pulses and SNR = 9.9. Figure 5 shows that the proposed method reduces the number of accumulated pulses, or the numbers of detected photons, to about a half as much as the matched filter method at the same ranging performance.

### 3.2. Experimental Results

**Figure 6:**
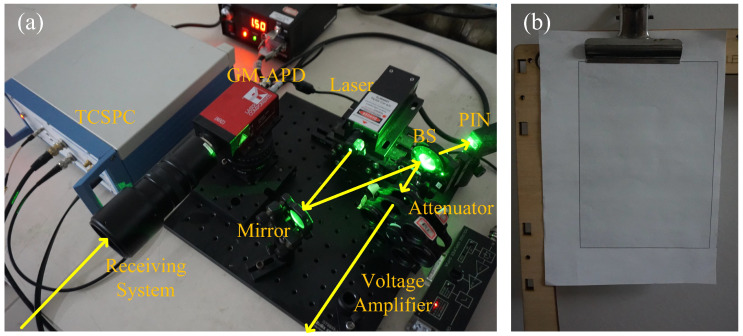
Photograph of the experimental setup. (**a**) The photon counting ranging system. (**b**) The target used in the experiment.

The photon counting ranging system shown in Figure 6 is established to experimentally demonstrate the proposed method. The experimental configurations are listed in Table 2. The system uses the pulsed laser to illustrate the target. A PIN detector (THORLABS, DET025A/M) equipped with a voltage amplifier (TEMTO, DHPVA-200) synchronizes the emitting laser pulses to generate the start signal to the time-correlated single photon counting (TCSPC) module. A Si SPAD (Laser Component, COUNT-100C) detects the echo photons captured by the receiving system (NAVITAR, ZOOM 7000) and generates avalanche responses. The TCSPC module (SIMINICS, FT1010) records the arrival time stamps and then builds a photon counting histogram. The target used in this experiment is a gray paper as shown in Figure 6b. Before the experiment, the number of signal photoelectrons reflected by the target was calibrated in the dark environment. The intensity of emitted laser was attenuated by the attenuator, and the number of echo photoelectrons is adjusted to about 0.05, which is consistent with the simulation conditions.

To verify the anti-noise performance of the proposed method, the ranging experiments under four different background noise levels are undertaken. The target range is calculated by Equations (11)–(13). In the experiment, the background noise level is set using a sunlight lamp, and 1024 times range measurements have been taken at each noise level. Each range measurement accumulates 1500 laser pulses to build an arrival time histogram. The SNRs under different background noise levels (3 MHz, 5 MHz, 7 MHz and 9 MHz) are respectively 6.9, 6.2, 5.6, and 5.2. In the experiment, the method of comparison is only the matched filter method, as the anti-noise performance of direct thresholding method is much poorer than that of the other two methods compared in the simulation using a weak signal but strong background noise.

**Figure 7:**
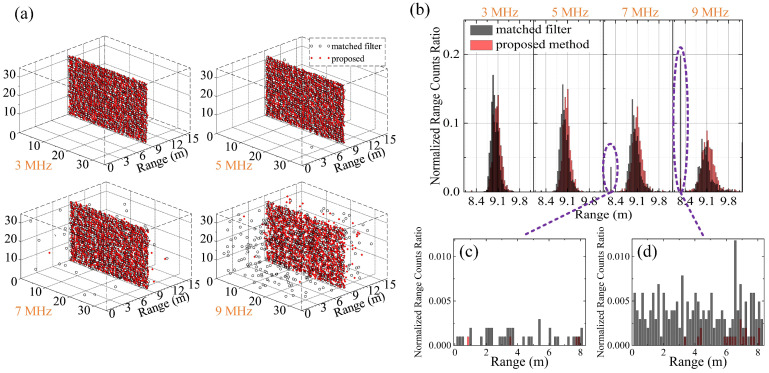
Experimental results of noise mitigation and ranging experiments. (**a**) The visualization of the measured ranges of the proposed method (red rhombus) and the matched filter method (black circle), each point represents a measured range value; (**b**) Range histogram corresponding to (**a**); (**c**) Range histogram between 0 and 8 m under 7 MHz background noise rate; (**d**) Range histogram between 0 and 8 m under 9 MHz background noise rate.

The experimental results of the proposed method are shown in Figure 7. Figure 7a shows the visualization of the measured ranges, in which each point is a range value. Figure 7b–d are the corresponding range histograms, whose vertical axis is the ratio of the measured ranges falling within each range interval to the number of repeated experiments. The range visualization of each noise level is composed of 32 × 32 measured ranges. Figure 7a shows that, as the background noise increases, the measured range values of the proposed method are always concentrated and distributed near the true range, whereas those of the matched filter method become obviously scattered and are distributed away from the true range. Specifically, at a background noise of 9 MHz, as is shown in the Figure 7b,d, a large number of measured range values obtained by matched filter fall into the intervals in front of ∼9.1 m, resulting in poor noise robustness and ranging performance.

### 3.3. Discussion on Experimental Results

The quantitative performance of the experimental results in Figure 7 for comparison is calculated using Equations (14)–(16) and listed in Table 3. For ranging error performance, it is consistent with the experimental results. When the background noise rate is 9 MHz, range accuracy and range precision of the proposed method are 27.8 cm and 56.2 cm, and those of the matched filter method are 187.2 cm and 330.6 cm, respectively. It is about five times that of the range accuracy and range precision decrease. For noise robustness, the proposed method gives correct range estimations more frequently than the matched filter. The correct estimation rate of the former is about 20% better than the latter at 9 MHz noise level. Therefore, the proposed method mitigates the effect of background noise effectively with more robustness than the matched filter method, which is helpful to reduce the ranging error under high background noise.

**Figure 8:**
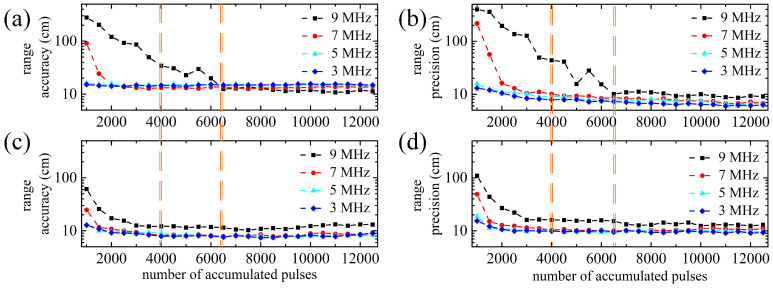
Influence of the number of accumulated pulses on ranging error of the two methods under different noise levels: (**a**) Range accuracy of the matched filter method; (**b**) Range precision of the matched filter method; (**c**) Range accuracy of the proposed method; (**d**) Range precision of the proposed method. The number of accumulated pulses is increasing from 1000 to 12,500 with a step of 500.

Then, the influence of the number of accumulated pulses on the ranging error is discussed. Figure 8 shows the results of the ranging error varying with the number of accumulated pulses under different noise levels. In Figure 8, the trend of range accuracy and range precision of the two methods is similar. At low background noise levels such as 3 MHz and 5 MHz, both methods maintain range accuracy and range precision near stable values, but the matched filter method is slightly superior to the proposed method. When the background noise is strong, range accuracy and range precision of the proposed method converge rapidly to the stable value with the increase of the accumulated pulses, but those of the matched filter method converge slowly. At the background noise of 7 MHz, the proposed method requires only 1500 pulses and SNR of 5.6 to the stable range accuracy and range precision, ∼9.8 cm and ∼11.1 cm, respectively. The matched filter method needs about 3000 pulses and SNR of 8.0 to a comparable performance. At the background noise of 9 MHz, the proposed method requires only 4000 pulses with SNR of 8.5 while the matched filter needs at least 6500 pulses with SNR of 10.9. Under the same ranging performance, the proposed method reduces the number of accumulated pulses by 1 times at 7 MHz and 0.5 time at 9 MHz, and reduces the working SNR by more than 2, which is consistent with the simulations. Therefore, Table 3 and Figure 8 jointly verify that the proposed method can still deal with strong background noise despite lower SNR, and it has good performance of mitigating noise effect to reduce ranging error in the case of high background noise robustly.

## 4. Conclusions

In conclusion, an entropy-based anti-noise method is proposed to robustly mitigate the effect of background noise in photon counting lidar, so as to reduce the ranging error. The photon counting entropy has been defined firstly to evaluate the uncertainty of the fluctuation among PEs responded by Gm-APD and used as the feature to distinguish signal from noise. Then combined with a window operation, noise was dealt with by replacing the raw photon counting data with the sequence of the photon counting entropy. Finally the time of flight is estimated by searching for the subinterval with minimum photon counting entropy. Simulation has analyzed that the proposed method has better anti-noise performance under various noise levels than the matched filter method. Experimental results have demonstrated that the proposed method effectively mitigates noise to reduce the ranging error despite a weak signal and high background noise. For example, under a background noise rate of 9 MHz, range accuracy and range precision of the proposed method are about five times better than the matched filter method. Besides, the proposed method requires less detected photons and lower SNR, i.e., the proposed method reduces the number of accumulated pulses by 0.5 times and the working SNR by 2 compared with matched filter method, at the same level of ranging performance. This method does not increase the system complexity, and may be applied to long-range and full-time detection of small targets.

## Figures and Tables

**Table 1:** Simulation parameters. The number of the signal photoelectrons is ∼0.05 according to these parameters.

Module	Items	Parameters
laser	wavelength	532 nm
	repetition period	4 kHz
	pulse width	3.2 ns
	single shot energy	1 nJ
receiving system	transmittance	0.7
	aperture	5 cm
detector	quantum efficiency	35%
	dead time	45 ns
time correlator	time resolution	64 ps
	number of time bins	1024
target	reflectivity	0.1
	range	650 m
	signal location	760-th time bin

**Table 2:** Experimental configurations.

Module	Items	Parameters
laser	wavelength	532 nm
	repetition period	4 kHz
	pulse width	4 ns
	single shot energy	5 μJ
receiving system	transmittance	0.79
	aperture	5 cm
detector	quantum efficiency	55%@532 nm
	dead time	45 ns
	dark counts rate	100 Hz
TCSPC	time resolution	64 ps
target	size	11cm×15.5cm
	range	9.3 m

**Table 3:** Performance of ranging error and noise robustness under various noise levels.

	Method	3 MHz	5 MHz	7 MHz	9 MHz
$\sigma_{\mathrm{accuracy}}$ (cm)	matched filter	14.4	15.5	25.6	187.2
	proposed	10.8	10.7	12.8	27.8
$\sigma_{\mathrm{precision}}$ (cm)	matched filter	11.3	13.1	68.3	330.6
	proposed	12.4	12.4	16.9	56.2
$r_{c}$	matched filter	0.999	0.994	0.925	0.661
	proposed	0.998	0.999	0.981	0.891

**Figure A1:**
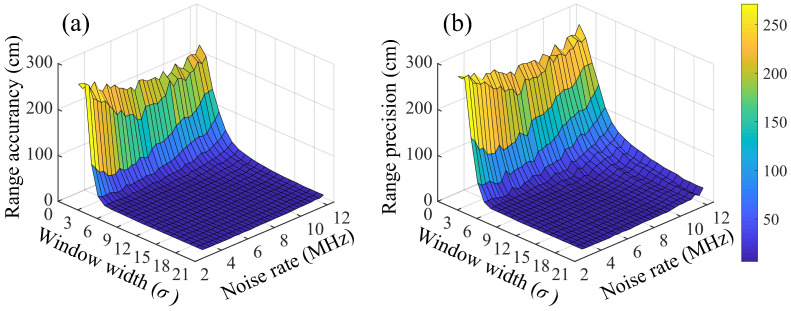
The influence of window width on range accuracy and range precision under different noise levels. (**a**) Range accuracy under various window widths and background noise rates; (**b**) Range precision under various window widths and background noise rates.

## Data Availability

Data available on request due to privacy.

## Appendix A. Window Width Selection

This section aims to discuss the influence of window width on range accuracy and range precision under different noise levels, so as to provide reference for selecting a proper window width in this work. The simulation parameters are the same with Table 1. The simulation result is shown in Figure A1, in which the window width varies form 1σ to 20σ, and background noise rate ranges from 3 MHz to 12 MHz, where σ is the standard deviation of the Gaussian laser pulse. In Figure A1, better range accuracy and range precision are obtained with the window width increasing under different noise levels. For the noise level ∼10 MHz (regarding the solar background noise rate in daytime), range accuracy and range precision both decrease to acceptable values (∼20 cm) with a window width of 6σ∼7σ. As the window width continues to increase, range accuracy and precision are gradually stabilizing to a constant. However, considering that it is not conducive to separate two adjacent ranges when choosing larger window widths, and more importantly, a width of 6σ contains about 99% of the total energy of Gaussian laser pulse, the window width of 6.5σ with relaxation is selected.
